# Supplementary material for: The genome sequence of the pebble prominent, Notodonta ziczac (Linnaeus, 1758)
Source: Wellcome Open Res. 2022 Mar 25;7:111. [Version 1] doi: 10.12688/wellcomeopenres.17789.1 (PMC9171288; doi:10.12688/wellcomeopenres.17789.1)
Supplement: Peer review history

# Peer review: Reviewer response for version 1

**DOI:** 10.21956/wellcomeopenres.19689.r50553

**Published:** 2022-06-06

**Authors:** Christopher M. Ward

**Affiliations:** 1 Australian Wine Research Institute, Adelaide, SA, Australia

The data note by the late Boyes *et al., *describes the full genome sequence of *Notodonta ziczac *(Linnaeus, 1758). The data note is concise and in line with others of its kind, providing a lucid description of the organism and of the assembly. Therefore, I have no hesitation to support its indexing.

Are sufficient details of methods and materials provided to allow replication by others?

Yes

Is the rationale for creating the dataset(s) clearly described?

Yes

Are the datasets clearly presented in a useable and accessible format?

Yes

Are the protocols appropriate and is the work technically sound?

Yes

Reviewer Expertise:

Adaptation, insect-hostplant arms race, grapevine genetics

I confirm that I have read this submission and believe that I have an appropriate level of expertise to confirm that it is of an acceptable scientific standard.

# Peer review: Reviewer response for version 1

**DOI:** 10.21956/wellcomeopenres.19689.r49487

**Published:** 2022-05-26

**Authors:** Akito Y. Kawahara

**Affiliations:** 1 McGuire Center for Lepidoptera and Biodiversity, Florida Museum of Natural History, University of Florida, Gainesville, FL, USA

This is an important data note by the late Douglas Boyes and others. The species is in steep decline and there is thus an immediate impetus to make this genome assembly public. I see no problems with the way the paper is written or presented, and is in-line with many of the other genome notes. I fully support and approve the indexing of this Data Note.

Are sufficient details of methods and materials provided to allow replication by others?

Yes

Is the rationale for creating the dataset(s) clearly described?

Yes

Are the datasets clearly presented in a useable and accessible format?

Yes

Are the protocols appropriate and is the work technically sound?

Yes

Reviewer Expertise:

Lepidoptera evolution, genomics

I confirm that I have read this submission and believe that I have an appropriate level of expertise to confirm that it is of an acceptable scientific standard.
